# CONTeMPLATE—a mnemonic to help medical educators infuse reflection into their residency curriculum

**DOI:** 10.1007/s40037-017-0400-y

**Published:** 2017-12-18

**Authors:** Lawrence Cheung

**Affiliations:** Department of Medicine, Department of Critical Care, University of Alberta, Edmonton, AB Canada

**Keywords:** Curriculum development, Reflective practice, Residency education, Curriculum implementation

## Abstract

Reflection, where clinical experiences are analyzed to gain greater understanding and meaning, is an important step in workplace learning. Residency programs must teach their residents the skills needed for deep reflection. Medical educators may find it difficult to construct a curriculum which includes the key elements needed to enable learners to attain these skills. When we first implemented reflection into our residency curriculum, we soon realized that our curriculum only taught residents how to engage in superficial reflection. Our curriculum lacked some key elements. To help guide the transformation of our curriculum, we combed the literature for best practices. The CONTeMPLATE mnemonic was born out of this process. It is a tool to help medical educators consider and implement key elements required to enable deep reflection. The purpose of this article is to show medical educators how they can use the CONTeMPLATE mnemonic to incorporate reflective practice into their own curriculum.

## Background

Accreditation bodies stipulate that residency programs must nurture the knowledge, skills and attitudes for reflection in their residents. This includes understanding the stages of reflection, practising the cognitive skills needed to move through these stages, and instilling the attitude that reflection must be maintained throughout one’s career. Reflection can be defined as a deep analysis of experience to gain a greater understanding and meaning [1]. It is a key step in learning from clinical experiences [2].

Our two-year subspecialty respirology residency program accepts approximately three residents per year, each of whom have completed three years of core internal medicine residency. We have approximately six residents in the program at a time. When our residency program initially integrated reflection into our curriculum, we used the rubric developed by Wald et al. to assess our residents’ reflective writing [3]. We then realized that we were only teaching our residents to engage in superficial reflection. Similar to what has been observed in other training programs [4], our curriculum lacked some important elements. We failed to promote the skills needed for deeper reflection, neglected to assess reflection with appropriate tools, and did not provide sufficient, targeted feedback on the residents’ performance.

Through several cycles of planning, implementation, and revision, we gradually evolved our curriculum. During this transformation, we developed the CONTeMPLATE mnemonic as a tool to guide our curriculum’s restructuring. The nine components of our CONTeMPLATE framework are Competencies, Organization, Narrowed focus, Teaching, Methods, Prompts, Longitudinal assessment, Tailored feedback, and Environment. Other curriculum development frameworks [5] also outline basic elements needed for curriculum development. The advantage of our tool is that it tailors those basic elements to meet the specific needs of teaching a reflective curriculum. We selected our nine elements through an iterative process. The framework we developed can save medical educators time from having to adapt a generic model for their reflective curriculum. By focusing on more than just the method of reflection, our framework can help medical educators create a system of learning for reflective practice.

This article further describes each component of the CONTeMPLATE mnemonic and shows medical educators how they can use it to implement reflection into their own curriculum. We illustrate this by demonstrating how we used this mnemonic tool in practice.

## The CONTeMPLATE curriculum

The elements of the CONTeMPLATE mnemonic are summarized in Tab. 1. These elements are further discussed below.

**Table 1:** Components of the CONTeMPLATE Curriculum

*C*ompetencies	Identify the competencies required for reflection and write them as learning objectives
*O*rganization	Organize a regular time, place, and process for reflection to occur
*N*arrowed focus	Provide a starting point for reflection by narrowing the topics and splitting reflective exercises into themes
*Te*aching	Teach the knowledge, skills, and attitudes needed for reflection
*M*ethods	Choose a method which the residents will use to reflect
*P*rompts	Provide prompts in the form of questions to stimulate reflection
*L*ongitudinal *A*ssessment	Assess reflection longitudinally at multiple points over time
*T*ailored feedback	Provide tailored, specific feedback on the learners’ reflective skills
*E*nvironment	Promote the hidden curriculum by providing an environment for reflection and encouraging everyone to engage in this activity

### Competencies

When integrating reflection into the curriculum, medical educators must first establish the competencies that learners must acquire. Competencies consist of knowledge, skills, and attitudes. The goals of the curriculum are to help the residents attain these competencies, and the curricular objectives are written to express these goals.

Knowledge-based competencies include understanding the cognitive processes that occur during reflection. Atkins and Murphy [6] divided these cognitive processes into three main stages. These stages include identifying uncomfortable feelings or thoughts after an event, analyzing these feelings, then developing a new perspective after rethinking current knowledge.

Skills-based competencies include attaining the requisite skills to move through these stages of reflection. Atkins and Murphy identified five skills—self-awareness, description, critical analysis, synthesis, and evaluation—to achieve this task.

Attitude-based competencies include gaining the insight that reflection is vital for continuous professional learning. This is important so that residents continue to reflect throughout their medical career.

For our curriculum, we wrote objectives that described attainment of these competencies. For example, for self-awareness, our learning objective stated that ‘The resident will identify how the situation affected him/her and how his/her behaviour affected the situation’.

### Organization

After establishing the competencies and objectives, educators must organize the learning. Residents may superficially reflect on experiences as they happen. However, they may be preoccupied with their clinical workload and lack the time for deeper reflection [1]. Residents usually desire structured teaching for reflection [7]. The curriculum should include a time, a place, and guidance for this reflection to occur. Throughout the year, we allotted approximately one hour every three weeks for the residents to discuss their written reflections amongst their peers, led by a faculty facilitator. Before each of these small-group sessions, residents produced written narratives of their reflections. We scheduled a room suited for small-group discussion, and booked time—usually one to two hours—for this to occur. During these sessions, we freed the residents from their clinical duties. By regularly incorporating discussion of the residents’ reflection into their academic schedule, our residency program explicitly emphasized the importance of practising this skill.

### Narrowed focus

It helps if learners narrow the focus of their reflection. This provides a starting point for the process and makes the experience less overwhelming. Our residency program aligned our educational content along the CanMEDS framework [8]. Thus, it was natural for us to narrow the focus of reflection along these CanMEDS roles. For example, the theme of one session involved residents reflecting on a personal experience where conflict occurred amongst members within the healthcare team. The residents narrowed the focus of their reflection to the CanMEDS roles of communication, collaboration, and professionalism that arose within the situation.

### Teaching

As with other skills, we cannot assume that residents are intrinsically proficient in deep reflection [9]. They benefit from being shown and taught how to engage in this process. Also, faculty must be taught how to facilitate and model deep reflection because they may feel uncomfortable teaching and demonstrating this skill [10]. We first oriented our clinician-facilitators to the principles of deep reflection [11] and showed them how to teach this skill to our residents. Then, these clinician-facilitators led seminars for the residents. Residents were taught how to engage in reflection by using specific clinical situations drawn from their own experiences. In these seminars, the clinician-facilitators defined reflection, expounded the benefits of reflection to clinical practice and learning, distinguished superficial from deep reflection, and demonstrated the requisite skills.

### Methods

Different methods can be used to reflect. For example, reflection may be done through oral recitation or through writing. The process may be recorded on video or in reflective logs, journals, or portfolios. Korthagen and Vasalos’s ‘onion model’ deals with the contents of reflection—such as values and identity—and can also be used to enhance core reflection [12]. In our case, we asked our residents to write down their reflections using a method known as significant event analysis [13]. Before each small-group discussion with the other residents and faculty facilitator, residents reflected on a critical event they experienced in the clinical setting. The theme of the critical event would vary between sessions. Themes included providing care to dissatisfied or angry patients and their families, handling medical uncertainty, and working within a healthcare team. Residents would select a significant event they experienced along that theme, work through the stages of reflection, and write down their reflections. Then, during the small-group discussion, the residents would present their reflective writing. Other residents and the faculty facilitator would add their insights based on their own experiences, providing different perspectives. The facilitator would also guide the discussion.

### Prompts

Like signposts along a road, residents benefit from prompts as they adopt the skills enabling deep reflection. These prompts are questions about their experience that residents must ask themselves. They direct the residents towards the details on which they should focus their reflection. Too many prompts can overemphasize quantity at the expense of quality. Conversely, progression through the stages of reflection can stall if there are too few prompts. We used the prompts found in the LEaP Guide for Critical Reflection by Aronson et al. [4]. These prompts include considering the content, process, and premise of the experience, reconsidering the experience from multiple perspectives, and synthesizing the learning.

### Longitudinal assessment

Reflection is an iterative process—it must be repeatedly practised. Therefore, educators should conduct longitudinal assessment of this process. This requires the use of an objective tool to assess the learners’ reflection and evolution of their reflective skills. We used the REFLECT rubric—a qualitative, formative assessment tool by Wald et al. [3]—to assess our residents’ reflective writings.

### Tailored feedback

Tailored feedback helps improve the residents’ skills for deep reflection [14]. Feedback that is posed as a question, has a positive tone, and is adjusted to the learners’ reflective level can enhance reflective skills and stimulate ongoing reflection [15]. After the group session, faculty facilitators assessed each resident’s written reflection. Faculty then gave written formative feedback using the REFLECT rubric and discussed this feedback with the resident. During this discussion, the residents processed the feedback.

The REFLECT rubric already contains a structured approach for giving individualized feedback while allowing our facilitators some discretionary leeway in their responses. Alternatively, medical educators can use the BEGAN framework by Reis et al. [16], another useful tool for providing feedback on the learners’ reflective writing.

### Environment

Reflection must be woven into the environment of the residency program to reinforce this hidden curriculum. This inspires the attitude competency for reflective practice. In other words, it instils the attitude that reflective practice is a vital component for continuous professional learning in one’s medical career. Faculty must genuinely value reflection and explicitly demonstrate it in the day-to-day clinical setting. However, they may feel uncomfortable modelling reflection [17] and, like the residents, may lack the skills for deeper reflection. To increase reflection amongst our faculty, we started with our facilitators. All our facilitators were clinicians. We oriented them on the utility of reflection in clinical practice and the principles of modelling reflection. Then, gradually, they helped other faculty develop these skills through one-on-one sessions and small-group seminars. Learning goals included understanding the stages of reflection and practising the skills needed to move through these stages. Methods included the use of reflective writing. Faculty would write down their reflections. Then, using the REFLECT rubric, they would assess each other’s reflective narratives as well as sample narratives from the residents. We are in the process of formalizing this through increased peer review of teaching.

## Curriculum evaluation

For our small, subspecialty residency program, we evaluated our curriculum with feedback questionnaires from twelve residents and three clinician-facilitators over a span of three years. Overall, feedback from both groups was quite favourable. Residents felt that the curriculum enhanced their skills in reflective thinking. They thought the facilitators were knowledgeable, well prepared, demonstrated the skills for reflection, and delivered useful feedback on the reflective writing. Facilitators felt more comfortable modelling reflection and using reflection to further their own continuous professional learning.

In the future, we seek to improve our curriculum evaluation in two ways. First, it is possible that the residents simply wrote what they thought the facilitators wanted to see. We plan to survey the residents several years after they have become independent practitioners to determine if they have maintained their reflective practice. Second, we plan to evaluate our facilitators more comprehensively using the rating scale developed by Schaub-de Jong et al. [18]. This will assess the facilitators on the three domains of reflective teaching which include supporting self-insight, creating a safe environment, and encouraging self-regulation.

## Conclusion

Medical educators should use a structured approach when devising a curriculum. This principle holds true when creating a curriculum to teach learners the skills, attitudes, and behaviours for reflective practice. The CONTeMPLATE mnemonic is a tool that medical educators can use to create a system of learning for a reflective practice curriculum. Further work can determine if using this tool on large educational programs leads to successful outcomes.
